# Sleep Domain Predictors of Headache-Related Disability in Episodic Migraine and Cluster Headache: A Prospective Observational Cohort Study

**DOI:** 10.3390/jcm15072710

**Published:** 2026-04-03

**Authors:** Şenay Aydın, Suna Aşkın Turan

**Affiliations:** 1 Department of Neurology, University of Health Sciences, Yedikule Chest Disease and Surgery Training and Research Hospital, Istanbul 34020, Türkiye; 2 Pain Department, University of Health Sciences, Mersin City Research and Training Hospital, Mersin 33240, Türkiye; sunaaskin1@gmail.com

**Keywords:** episodic migraine, episodic cluster headache, sleep quality, insomnia, daytime sleepiness, sleep hygiene, headache-related disability

## Abstract

**Background:** Sleep disturbance is a well-recognized contributor to headache burden, yet the specific sleep domains associated with disability may differ between episodic migraine (EM) and episodic cluster headache (ECH). **Methods:** In this prospective observational study, 20 EM patients, 21 ECH patients, and 18 age-, sex-, and BMI-matched healthy controls (HCs) were evaluated during interictal periods. None of the patients were receiving prophylactic headache treatment. Sleep was assessed using the Pittsburgh Sleep Quality Index (PSQI), Insomnia Severity Index (ISI), Epworth Sleepiness Scale (ESS), and Sleep Hygiene Index (SHI). Psychological status was measured with the Hospital Anxiety and Depression Scale (HADS). Headache-related disability was assessed using the Headache Impact Test-6 (HIT-6) as a continuous outcome. Separate multivariable linear regression models were constructed for each headache group. **Results:** Both headache groups showed significantly impaired sleep and higher anxiety and depression scores compared with controls (all *p* < 0.001). HIT-6 scores did not differ between EM and ECH (*p* = 0.770 after Bonferroni correction). In multivariable regression, excessive daytime sleepiness (ESS) independently predicted disability in EM (B = 1.633, *p* = 0.033; R^2^ = 0.571). In ECH, global sleep quality (PSQI; B = 0.701, *p* = 0.004) and sleep hygiene (SHI; B = 0.557, *p* = 0.033) were independently associated with HIT-6 (R^2^ = 0.562). No significant multicollinearity was observed (all VIF < 2.5). **Conclusions:** Sleep disturbance is prevalent in both EM and ECH; however, the sleep domains associated with disability differ between phenotypes. Daytime sleepiness is more relevant in EM, whereas global sleep quality and sleep hygiene are more strongly associated with disability in ECH. These findings support a phenotype-specific approach to sleep assessment in headache management.

## 1. Introduction

Headache disorders are among the leading causes of years lived with disability worldwide. Migraine, a chronic neurological disorder characterized by attacks varying in severity, duration, and various neurological symptoms, and the second most common type of primary headache is classified as episodic and chronic. Episodic migraine (EM) is characterized by headaches that occur on fewer than 15 days per month according to the *International Classification of Headache Disorders, Third Edition* (ICHD-3, 2018) diagnostic criteria [1]. In comparison, cluster headache, which is observed less frequently, is a primary headache disorder characterized by severe, unilateral pain around the eye and ipsilateral autonomic cranial symptoms, negatively affecting daily life. These attacks, characterized by sudden and intense pain, recur once a year to once a day and occur 1 to 8 times a day; each attack lasts between 15 min and 3 h. Cluster headache is also divided into two different forms according to the duration of pain-free periods between attacks: episodic and chronic; in the episodic form, pain-free periods last longer than 3 months. Migraine is a prevalent condition, affecting approximately 11.6% of the general population, with rates of 11.4% in Europe and 16.4% in Turkey, where it is relatively slightly more prevalent [2,3]. Cluster headache has a recorded prevalence of 0.1–0.2% in the general population [4,5], and chronic cluster headache occurs in approximately 10–15% of individuals [5]. Furthermore, EM accounts for 50.8% of rare headaches [6]. Migraine and cluster headache are associated with severe and recurrent attacks that substantially impair quality of life [7,8,9]. Identification and modification of triggers—including lifestyle factors such as sleep, diet, and physical activity—are integral components of headache management [10,11,12,13].

Sleep disturbance has emerged as a clinically relevant and modifiable contributor to headache burden [14,15,16]. The neurobiological substrates linking sleep and headache differ markedly between these two disorders. In episodic migraine, insomnia, restless legs syndrome, and sleep-disordered breathing are frequently reported comorbidities; sleep fragmentation lowers the cortical excitability threshold and facilitates trigeminovascular activation [17,18]. In episodic cluster headache, the relationship with sleep is more intrinsically circadian: attacks characteristically occur during rapid eye movement (REM) sleep, and the disorder exhibits striking diurnal and circannual periodicity, implicating hypothalamic and suprachiasmatic nucleus dysfunction, with disruption of melatonin secretion and orexinergic signalling playing central roles [19,20,21,22,23]. These mechanistic differences suggest that the impact of sleep disturbance on headache-related disability may manifest differently across the two conditions—a distinction with important implications for targeted management [24,25,26].

The objective of this prospective observational cohort study was to therefore comprehensively characterize and compare multidimensional sleep profiles in patients with EM and ECH relative to healthy controls and to identify the sleep-related predictors of headache-related disability within each group. We hypothesized that, although sleep disturbance would be more prevalent in both headache groups compared to controls, the specific sleep domain most strongly associated with disability would differ between EM and ECH, reflecting their distinct neurobiological substrates.

## 2. Materials and Methods

### 2.1. Study Design and Ethical Approval

This prospective observational cohort study was performed at the Neurology Outpatient Clinic of a University-affiliated Training and Research Hospital over a one-year period, from 1 January 2024 to 1 January 2025. A prospective observational design was employed, as the research question necessitated longitudinal interictal assessment and the concurrent use of validated questionnaire batteries. Reporting adhered to the Strengthening the Reporting of Observational Studies in Epidemiology (STROBE) guidelines. The study protocol received ethical approval from the institutional Clinical Research Ethics Committee (Approval No: 2023/466; Date: 28 December 2023). Written informed consent was obtained from all participants prior to enrolment, and the study was conducted in full compliance with the Declaration of Helsinki.

### 2.2. Participants

All patients were assessed by the same neurologist (Ş.A.) with subspecialty expertise in headache and sleep disorders at a single structured outpatient visit during a confirmed interictal period. Consecutive adult patients aged 18–74 years attending the Neurology Outpatient Clinic with a confirmed diagnosis of episodic migraine (EM) or episodic cluster headache (ECH) were considered for inclusion. The above age range was selected to exclude pediatric headache phenotypes and patients with high comorbidity burden at extreme ages.

Based on the *International Classification of Headache Disorders, Third Edition* (ICHD-3) criteria, we included EM patients with headaches occurring less than 15 days a month and ECH patients with headaches occurring once a year to once a day, recurring 1 to 8 times a day, with each attack lasting between 15 min and 3 h, and pain-free periods lasting longer than 3 months [1]. The headache disorder must have been present for at least one year prior to enrolment to ensure diagnostic stability and exclude new presenting cases. It is established that depressive mood changes are more prevalent in headache patients compared to the general population; in addition, during headache attacks, individuals’ mood, anxiety levels, and concentration can be severely impacted, and behavioural changes may also occur [27,28]. Therefore, to avoid acute-phase confounding of sleep and disability assessments, patients were enrolled only during an interictal period—defined as the absence of a headache episode in the preceding 48 h for EM and the absence of an active cluster period for ECH.

Patients were excluded if they presented with any of the following: a previously diagnosed primary sleep disorder (e.g., obstructive sleep apnoea, narcolepsy, or restless legs syndrome); cognitive impairment; major psychiatric comorbidity (e.g., schizophrenia or bipolar disorder); significant systemic or neurological illness; a history of head or neck trauma; current substance misuse; or high-risk populations such as pregnant women. Chronic pain-related illnesses are closely correlated with sleep disturbances. The results of a systematic review and meta-analysis of patients with chronic non-cancerous pain highlighted that poor sleep is one of the most common symptoms in chronic pain conditions [29,30]. Therefore, individuals with any chronic illness or chronic pain condition other than headache were excluded from the study. Patients receiving any prophylactic headache treatment—including beta-blockers, calcium channel blockers, antiepileptic drugs, or tricyclic antidepressants—were excluded, as these agents may alter sleep architecture and autonomic function, thereby confounding sleep-domain assessments. Similarly, patients currently receiving antidepressant or anxiolytic pharmacotherapy were excluded. Patients meeting criteria for chronic migraine (headache on ≥15 days per month for more than three months) or chronic cluster headache were also excluded. Medication overuse headache, defined as analgesic use on more than 10 days per month for at least three months, served as an additional exclusion criterion.

**Figure 1:**
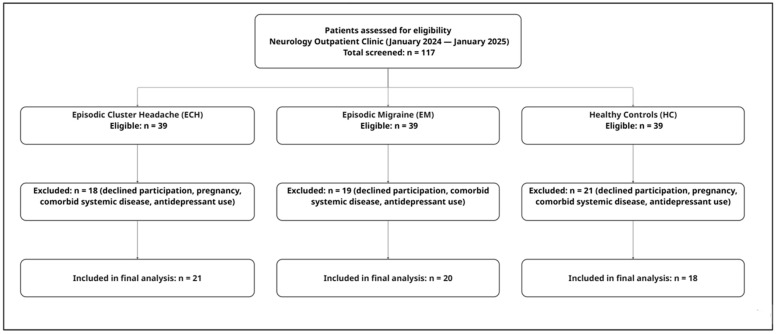
Patient flowchart.

A healthy control (HC) group was recruited from volunteers attending the same institution for routine medical review. Controls were individually matched to headache patients by age (±5 years), sex, and body mass index (BMI ± 2 kg/m^2^). Eligible controls had no history of chronic headache disorder and reported fewer than three headache episodes per month, none of which required pharmacological treatment. All exclusion criteria applied to the headache groups were applied equally to controls. The participant flow diagram is shown in Figure 1.

### 2.3. Data Collection and Outcome Measures

All data were collected via structured face-to-face interviews administered by the same neurologist (Ş.A.) to maintain consistency throughout the study. A standardized case report form was used to document sociodemographic variables, clinical headache characteristics (attack frequency, duration, pain quality, laterality, and associated symptoms) obtained from patient-maintained headache diaries and interviewer-administered headache assessment questionnaires, and current medication use. The following validated, Turkish-adapted questionnaires were administered to all participants [31,32,33,34,35,36].

Headache-related disability—the primary outcome measure—was assessed using the Headache Impact Test-6 (HIT-6; score range 36–78), with higher scores indicating greater disability [31]. HIT-6 was used as a continuous dependent variable in all regression analyses; a score above 60 was pre-specified as the threshold for severe impact for descriptive purposes. HIT-6 was selected over the MIDAS (Migraine Disability Assessment) questionnaire because it captures broader functional impact beyond lost days and is more sensitive to change.

Sleep was assessed across four independent domains: global sleep quality using the Pittsburgh Sleep Quality Index (PSQI; score range 0–21; score > 5 indicates poor sleep quality) [32] as the gold-standard multidimensional sleep quality measure; insomnia severity was dimensionally assessed using the Insomnia Severity Index (ISI; score range 0–28; score ≥ 15 indicates moderate-to-severe clinical insomnia) [33]; excessive daytime sleepiness (EDS) using the Epworth Sleepiness Scale as the standard clinical tool for daytime sleepiness (ESS; score range 0–24; score ≥ 10 indicates clinically significant EDS) [34]; and sleep-related behavioural practices using the Sleep Hygiene Index (SHI; score range 13–65; higher scores reflect poorer sleep hygiene) [35].

Psychological status was assessed using the Hospital Anxiety and Depression Scale (HADS), which includes seven-item subscales for anxiety (HADS-A) and depression (HADS-D), each scored from 0 to 21. Subscale scores of 8 or higher indicate probable clinical caseness [36]. The HADS was used because it was specifically developed and validated for medically ill populations, avoiding somatic symptom confounding. Healthy controls completed the same questionnaire battery, excluding the HIT-6, which was not applicable without a headache diagnosis.

### 2.4. Statistical Analysis

Given the rarity of episodic cluster headache in outpatient neurology settings, this study was designed as an exploratory, hypothesis-generating investigation. Although no a priori power calculation was performed, a post hoc power estimation indicated that the achieved statistical power was approximately 0.70–0.80 for detecting medium-to-large effect sizes (β = 0.522 for ESS in EM; β = 0.604 for PSQI in ECH) at α = 0.05, supporting the reliability of the primary significant findings. Post hoc power analysis was conducted using G*Power 3.1 software, applying the observed standardized regression coefficients (β = 0.522 for ESS in EM; β = 0.604 for PSQI in ECH) at α = 0.05 with the achieved sample sizes [37]. All findings should nonetheless be considered preliminary and require confirmation in larger multicentre cohorts.

Continuous variables such as age, body mass index, PSQI, ESS, ISI, SHI, HADS scores, and HIT-6 scores are summarized as mean ± standard deviation (SD); categorical variables such as sex, smoking status, alcohol use, marital status, education level, and headache symptoms are reported as frequencies and percentages. Normality of distribution was assessed using the Shapiro–Wilk test, and given that most variables were not normally distributed, non-parametric tests were preferred. For comparisons among the three groups (episodic migraine, episodic cluster headache, and healthy controls), the Kruskal–Wallis test was used for continuous variables; for example, PSQI, ESS, ISI, SHI, HADS-Anxiety, and HADS-Depression scores were compared among the three groups using this test. When a significant difference was detected (e.g., PSQI *p* < 0.001, ESS *p* < 0.001), pairwise comparisons were performed using the Mann–Whitney U test with Bonferroni correction. Categorical variables such as sex, smoking, alcohol use, tea/coffee consumption, marital status, employment status, and education level were compared using the chi-squared test or Fisher’s exact test as appropriate, with Fisher’s exact test applied when any expected cell frequency was less than five. For the two-group comparison of headache characteristics (EM vs. ECH), the Mann–Whitney U test was used for continuous variables such as headache days per month, attack duration, and disease duration, while categorical variables including unilateral pain, pulsating pain, stabbing/boring pain, photophobia, phonophobia, nausea/vomiting, cranial autonomic symptoms, and agitation/restlessness were compared using the chi-squared or Fisher’s exact test as appropriate.

To examine associations between sleep domains and headache-related disability, separate multivariable linear regression models were constructed for the EM and ECH groups, with HIT-6 score entered as a continuous dependent variable. All theoretically relevant sleep predictors (PSQI, ESS, SHI, and ISI) and HADS-Anxiety were entered simultaneously into each model to avoid selective variable inclusion; no stepwise or automated selection procedures were used. The analysis yielded unstandardized regression coefficients (B), standard errors (SEs), standardized coefficients (β), t-statistics, 95% confidence intervals (CIs), and overall model fit assessed via the coefficient of determination (R^2^). Multicollinearity was assessed using the variance inflation factor (VIF); all predictors demonstrated acceptable VIF values (<2.5), indicating limited predictor redundancy. Statistical significance was set at *p* < 0.05. All analyses were performed using IBM SPSS Statistics, version 22 (IBM Corp., Armonk, NY, USA).

## 3. Results

### 3.1. Sociodemographic Characteristics

A total of 59 participants were enrolled: 20 with EM (mean age 33.0 ± 6.8 years; 70.0% female), 21 with ECH (mean age 32.3 ± 6.8 years; 42.9% female), and 18 healthy controls (mean age 36.7 ± 7.7 years; 55.6% female). The participants’ sociodemographic characteristics are shown in Table 1. The three groups were well matched for age (*p* = 0.164), sex (*p* = 0.216), BMI (*p* = 0.413), smoking (*p* = 0.182), and alcohol use (*p* = 0.114). Two between-group differences reached statistical significance: single marital status was most prevalent in the ECH group (66.7%) and least prevalent in the EM group (15.0%; *p* = 0.006), and educational attainment differed significantly across groups (*p* = 0.003), with both groups exhibiting higher attainment than the healthy population, particularly in the EM group, wherein a greater proportion were educated to primary or secondary level; in comparison, university education was more prevalent in the EHC group. These imbalances were identified as potential confounders; however, they could not be included as covariates in the regression models due to sample size constraints.

### 3.2. Headache Characteristics

Headache characteristics are summarized in Table 2. Patients with EM experienced significantly more headache days per month (9.3 ± 3.2 vs. 6.6 ± 4.3; *p* = 0.044) and much longer individual attack duration (27.5 ± 31.4 h vs. 1.8 ± 0.9 h; *p* < 0.001). Pulsating pain quality predominated in EM (70.0%), while stabbing or boring pain predominated in ECH (66.7%; *p* = 0.001). Unilateral pain was more prevalent in EM (75.0% vs. 33.3%; *p* = 0.018). Cranial autonomic symptoms were present in 95.2% of ECH patients compared with 15.0% of EM patients (*p* < 0.001). Photophobia (75.0% vs. 9.5%), phonophobia (80.0% vs. 9.5%), and nausea or vomiting (80.0% vs. 4.8%) were all significantly more frequent in EM (all *p* < 0.001). Agitation or restlessness during attacks was reported by 57.1% of ECH patients compared with 5.0% of EM patients (*p* < 0.001). Disease duration did not differ significantly between groups (56.1 ± 89.5 vs. 27.4 ± 30.4 months; *p* = 0.187).

### 3.3. Sleep Parameters, Daytime Sleepiness, and Psychological Status

Sleep and psychological parameters are presented in Table 3. Both headache groups exhibited significantly worse sleep profiles than healthy controls across all four sleep domains (Kruskal–Wallis, all *p* < 0.001). ISI scores indicating moderate-to-severe insomnia were observed in both headache groups (EM: 19.4 ± 3.4; ECH: 24.7 ± 1.8) compared with controls (6.2 ± 3.0); post hoc testing confirmed that ECH patients experienced significantly more severe insomnia than EM patients (*p* < 0.001). ESS scores exceeded the excessive daytime sleepiness threshold in both headache groups (EM: 11.1 ± 2.1; ECH: 16.9 ± 2.6; HC: 7.6 ± 3.0), with ECH patients showing significantly greater sleepiness than both EM patients (*p* < 0.001) and controls (*p* < 0.001). Sleep hygiene was most impaired in ECH (SHI: 25.7 ± 3.1), followed by EM (21.7 ± 3.8) and controls (14.3 ± 2.7), with all pairwise comparisons significant. PSQI scores exceeded the poor sleep quality threshold in both headache groups (EM: 9.2 ± 4.4; ECH: 9.9 ± 3.0; HC: 5.3 ± 2.1), with no significant difference between the two headache groups (*p* = 1.000 after Bonferroni correction). HIT-6 scores did not differ between the two headache groups (50.1 ± 6.6 vs. 49.7 ± 3.5; *p* = 0.770 after Bonferroni correction), indicating comparably high disability burdens.

HADS-Anxiety was significantly higher in both headache groups compared with controls (EM: 13.8 ± 3.9; ECH: 17.0 ± 2.2; HC: 9.2 ± 1.7; *p* < 0.001), with ECH patients experiencing the greatest anxiety burden. Pairwise comparisons were significant for EM vs. HC (*p* < 0.001), ECH vs. HC (*p* < 0.001), and EM vs. ECH (*p* = 0.025). HADS-Depression was also elevated in both headache groups relative to controls (EM: 15.3 ± 4.3; ECH: 14.9 ± 3.3; HC: 8.0 ± 1.6; *p* < 0.001), with no significant difference between the two headache groups (*p* = 1.000 after correction).

### 3.4. Predictors of Headache-Related Disability: Multivariable Linear Regression

Results of the multivariable linear regression analyses are presented in Table 4. Both models were statistically significant and explained substantial variance in HIT-6 scores.

In the EM group (R^2^ = 0.571, model *p* = 0.023), excessive daytime sleepiness (ESS) was the only independent predictor of HIT-6 score to reach statistical significance (B = 1.633, SE = 0.691, β = 0.522, t = 2.362, *p* = 0.033; 95% CI: 0.150–3.116): each one-point increase in ESS score was associated with a 1.633-point increase in HIT-6. No other variable was a significant predictor in the EM model (PSQI: *p* = 0.990; SHI: *p* = 0.837; ISI: *p* = 0.974; HADS-Anxiety: *p* = 0.177).

In the ECH group (R^2^ = 0.562, model *p* = 0.019), the pattern of predictors differed markedly. Global sleep quality (PSQI) was the strongest independent predictor (B = 0.701, SE = 0.207, β = 0.604, t = 3.381, *p* = 0.004; 95% CI: 0.259–1.143), followed by sleep hygiene (SHI: B = 0.557, SE = 0.238, β = 0.485, t = 2.343, *p* = 0.033; 95% CI: 0.050–1.064). ESS, ISI, and HADS-Anxiety did not reach significance in the ECH model (ESS: *p* = 0.198; ISI: *p* = 0.228; HADS-Anxiety: *p* = 0.456).

These findings indicate that the sleep domain driving headache-related disability differs between conditions: daytime sleepiness in EM and global sleep quality combined with sleep hygiene behaviour in ECH.

### 3.5. Figures, Tables, and Schemes

Notably, ECH patients exhibited the most severe sleep disturbance across all domains, with significantly higher insomnia severity (ISI: 24.7 ± 1.8), excessive daytime sleepiness (ESS: 16.9 ± 2.6), and sleep hygiene impairment (SHI: 25.7 ± 3.1) compared with both EM patients and healthy controls; global sleep quality (PSQI) was similarly impaired in both headache groups relative to controls, with no significant difference between EM and ECH (*p* = 1.000), and HIT-6 disability scores did not differ between the two headache phenotypes (*p* = 0.770).

In the episodic migraine model, excessive daytime sleepiness (ESS) was the sole independent predictor of headache-related disability, with each one-point increase in ESS score associated with a 1.633-point increase in HIT-6 (β = 0.522, *p* = 0.033), accounting for 57.1% of the variance in disability scores (R^2^ = 0.571).

In the episodic cluster headache model, both global sleep quality (PSQI: B = 0.701, β = 0.604, *p* = 0.004) and sleep hygiene (SHI: B = 0.557, β = 0.485, *p* = 0.033) independently predicted headache-related disability, together explaining 56.2% of the variance in HIT-6 scores (R^2^ = 0.562), whereas daytime sleepiness, insomnia severity, and anxiety did not reach significance.

## 4. Discussion

The results of the present study demonstrate that while sleep disturbances are pervasive in both EM and ECH, the specific sleep modalities that independently predict headache-related disability differ fundamentally between these two disorders. Both headache groups exhibited significantly impaired sleep and elevated levels of anxiety and depression compared to healthy controls; however, despite these shared characteristics, the sleep domains associated with functional burden diverged clearly across phenotypes: daytime sleepiness in EM and global sleep quality combined with sleep hygiene behaviour in ECH.

To the best of our knowledge, this is one of the few studies in which multidimensional sleep profiles and their differential associations with disability are compared across these two distinct headache phenotypes within a unified prospective framework. The authors of previous studies have generally examined sleep disturbances in EM and ECH independently, with limited cross-phenotype comparison. For instance, Tiseo et al. (2020) conducted a systematic review confirming the high prevalence of sleep disorders in migraine but did not extend this analysis to cluster headache [18]; in addition, Gorgoni et al. (2024) reviewed objective sleep assessment specifically in ECH without migraine comparison [25]. The divergent disability-relevant sleep domains identified in the present study—daytime sleepiness in EM versus global sleep quality and hygiene in ECH—are consistent with the neurobiologically distinct mechanisms underlying these disorders and extend the existing literature by demonstrating that these differences translate into clinically meaningful, phenotype-specific predictors of functional burden. Data on the prevalence of ECH in the Turkish population remain limited. In a large-scale study conducted in a rural region, the annual prevalence among adults aged 14–65 years was reported as 0.04% [38]; among university students, this figure was comparatively higher at 0.2% [39]. From a Turkish clinical context, it is noteworthy that the migraine prevalence observed in our sample (16.4% nationally, as reported by Ertas et al.) exceeds the global estimate of approximately 11–12%, which may reflect differences in healthcare-seeking behaviour, stress burden, or cultural factors influencing symptom reporting in the Turkish population [3]. Furthermore, a lower male-to-female ratio was observed in our ECH cohort (approximately 1.4:1) compared to the internationally reported ratio of 3–4. The results of another study conducted in Turkey revealed a male-to-female ratio of 4:1 among individuals with ECH, highlighting a clear male predominance in this population [40]. In our study, examination of the gender variable revealed a female predominance in the EM group (70.0%), consistent with the literature; in comparison, the proportion of males was relatively higher in the EHC group (57.1%). This difference, however, did not reach statistical significance. This discrepancy with previous reports may reflect recruitment bias in a tertiary centre population, whereby higher educational attainment and greater health literacy may facilitate healthcare-seeking among women with cluster headache who might otherwise remain undiagnosed. In the present study, patients with EM experienced significantly more headache days per month and substantially longer individual attack durations compared with ECH patients (9.3 ± 3.2 vs. 6.6 ± 4.3 days; 27.5 ± 31.4 vs. 1.8 ± 0.9 h, respectively). These findings are consistent with previous reports indicating that migraine attacks are typically longer in duration and more frequent than cluster headache episodes [1]

Unilateral pain was significantly more common in EM (75.0% vs. 33.3%), which also corresponds with the results of prior studies emphasizing that migraine attacks are frequently lateralized; in comparison, cluster headaches, although often unilateral, may present with side-shifting in some cases [41]. Cranial autonomic symptoms were present in nearly all ECH patients (95.2%), compared with only 15.0% of EM patients. This finding is consistent with the diagnostic hallmark of cluster headache, in which ipsilateral autonomic features such as lacrimation, nasal congestion, and eyelid oedema are highly prevalent [42]. Photophobia, phonophobia, and nausea/vomiting were significantly more frequent in EM than ECH, which corroborates the well-established association of migraine with sensory hypersensitivity and gastrointestinal symptoms [43]. Conversely, ECH attacks were associated with agitation and restlessness in 57.1% of patients, consistent with prior observations that cluster headache patients tend to pace or exhibit motor restlessness during attacks, unlike migraine patients who often prefer to remain still [42]. Finally, disease duration did not differ significantly between the groups, suggesting that the chronicity of EM and ECH may not influence the frequency or severity of attacks in this cohort, in line with the prior literature in which heterogeneous disease courses are reported for both disorders [44]. Overall, the present findings reinforce key differentiating features between EM and ECH, including attack duration, pain quality, autonomic involvement, sensory sensitivity, and behavioural response during attacks, which are consistently demonstrated by the results of previous studies and remain clinically relevant for diagnosis and management.

Our results demonstrated that sleep parameters were significantly impaired in patients with EM and ECH compared to healthy controls. Furthermore, the higher scores in the ECH group compared to the EM group suggest that sleep-related mechanisms may be more prominent in cluster headaches. The strong association between primary headaches and sleep disturbances is well- established in the literature, and these findings are consistent with current studies. Sleep disturbances are an independent factor that can increase migraine severity [45]. Poor sleep quality is associated with migraine severity, and headache burden increases as PSQI scores rise [17]. In comparison, a significant proportion of cluster headache patients experience moderate-to-severe insomnia and serious sleep disturbances during active attacks, and this condition may not fully resolve even during remission [46]. Poor sleep hygiene and low sleep quality are common in primary headache patients. It has been reported that sleep disturbances can increase central sensitization, lower an individual’s pain threshold, and thus, this vicious cycle can contribute to the chronicity of headaches [47]. Both types of headaches are related to circadian rhythm and hypothalamic mechanisms, and therefore, disruption in sleep architecture may form part of the pathophysiology of the disease [23].

In the EM cohort, the ESS was the sole independent predictor of HIT-6 scores, suggesting that for individuals with migraine, the functional impact of the disease is not confined to nocturnal disruption alone but instead extends to its downstream consequence—impaired daytime alertness. This finding is consistent with the bidirectional sleep–migraine relationship documented in the literature, whereby sleep fragmentation lowers the threshold for trigeminovascular activation, generating a cycle of daytime impairment that amplifies migraine-related disability [15,18]. An additional explanatory mechanism may relate to the well-established behaviour of migraineurs seeking dark, quiet environments and napping during attacks; this practice, while acutely effective, may progressively disrupt circadian sleep–wake rhythmicity, perpetuating daytime sleepiness as an interictal phenomenon. Although this hypothesis warrants prospective investigation, it offers a plausible behavioural pathway linking attack-time sleep behaviour to interictal functional burden [24]. In the ECH group, disability was independently predicted by global sleep quality (PSQI) and sleep hygiene (SHI). The prominence of sleep hygiene as a predictor is particularly noteworthy, as it implicates irregular sleep scheduling and maladaptive sleep-related behaviours as amplifiers of the circadian dysfunction that is mechanistically central to ECH. Hypothalamic activation, suprachiasmatic nucleus disruption, and abnormalities in melatonin secretion and orexinergic signalling are well-established features of ECH pathophysiology [19,20,21,22,23]. Given that ECH attacks characteristically occur during REM sleep and exhibit striking diurnal and circannual periodicity, maintaining a stable sleep–wake cycle through adherence to consistent sleep hygiene practices may be particularly relevant for reducing functional impairment in this population [21,22].

Notably, although anxiety and depression were significantly elevated in both headache groups, they did not emerge as independent predictors of HIT-6 in either regression model. This finding indicates that sleep modalities have a direct, independent effect on headache-related disability that is not simply a consequence of psychological distress and that sleep-focused assessment and intervention targets are distinct from those addressing mood disorders. Supporting evidence in the literature demonstrates that sleep quality independently influences headache impact on quality of life even after adjusting for psychological factors such as anxiety and depression. For example, in a large clinic-based study of primary headache disorders, worse sleep quality measured using the PSQI was independently associated with higher HIT-6 scores in both migraine and tension-type headache after controlling for age, headache frequency, severity, and HADS anxiety/depression scores, indicating a direct effect of sleep quality on headache-related burden [48]. Although ECH differs in pathophysiology from migraine and tension-type headache, several studies highlight significant sleep disturbances in ECH that are not fully explained by anxiety or depressive symptoms. Patients report reduced sleep quality and difficulty coping with reduced sleep, with poor sleep quality and nocturnal attacks frequently observed even when adjusting for mood comorbidity [46]. Cross-sectional data demonstrate that sleep disturbances in cluster headache are linked to the attack cycle itself, including nocturnal onset patterns, which worsen sleep quality and can increase headache burden independently of anxiety or depression [46,49]. Although more studies are needed to directly examine the relationships between sleep quality, psychological factors, and headache impact (e.g., HIT-6) in ECH patients, current evidence suggests that sleep factors play a unique role in headache impact and should be assessed and managed separately from mood disorders [49].

From a clinical perspective, these findings suggest that routine sleep screening in headache clinics should extend beyond generic insomnia assessment and adopt a phenotype-specific approach. In EM, daytime sleepiness may serve as an accessible clinical marker of functional burden, warranting evaluation for fragmented sleep, delayed sleep phase, or behavioural sleep irregularities. In ECH, structured assessment of overall sleep quality and sleep hygiene may be particularly relevant given the disorder’s circadian basis. Incorporating brief, validated instruments such as the ESS or PSQI into standard outpatient evaluation requires minimal time and may identify patients at increased risk of functional impairment, thereby guiding timely behavioural or chronotherapeutic intervention.

## 5. Strengths and Limitations

This study has several strengths. First, we directly compare multidimensional sleep profiles across EM and ECH within a single methodological framework, minimizing inter-study heterogeneity. Second, all participants were evaluated during clearly defined interictal periods, limiting the confounding influence of acute attack-related factors. Third, the simultaneous use of four validated sleep instruments enabled comprehensive, domain-specific characterisation that would not be possible with a single global measure. Fourth, all theoretically relevant sleep domains and psychological covariates were entered simultaneously into the regression models, avoiding selective variable inclusion.

Several limitations should be acknowledged. First, the sample sizes within each headache subgroup were modest, increasing the risk of coefficient instability, reducing statistical power, and limiting the generalizability of the findings. Small subgroup numbers may render regression estimates sensitive to minor data variations and increase the likelihood of type II error. Therefore, the regression analyses should be considered exploratory rather than confirmatory, with replication in larger cohorts required. The limited sample size also restricted the number of covariates included in multivariable models, raising the possibility of residual confounding. The relatively short follow-up period further limits the assessment of the long-term stability of the observed associations. A brief observation interval may not capture delayed effects, temporal variability in headache severity, or long-term interactions between sleep disturbances and disability. Accordingly, the findings mainly reflect short-term relationships, with studies with longer longitudinal follow-up required to confirm their persistence and clinical relevance. In addition, several variables did not meet the assumption of normal distribution. Parametric analyses, including linear regression, rely on normally distributed residuals and homoscedasticity, and violation of these assumptions may affect the accuracy of standard errors, confidence intervals, and *p*-values. Although data transformations and non-parametric methods were applied when appropriate, these approaches generally have lower statistical power, particularly in small samples. Therefore, the combination of non-normal distribution and limited sample size may have reduced the sensitivity of the analyses and increased uncertainty in the estimated effects. Second, sleep parameters were assessed exclusively using validated self-report questionnaires; objective measurements such as actigraphy or polysomnography were not obtained, and subjective sleep perception may not fully capture physiological sleep architecture. Third, the cross-sectional design of the sleep–disability analysis precludes conclusions regarding directionality—sleep disturbance may contribute to disability, disability may impair sleep, or both may share common neurobiological factors.

Lastly, certain sociodemographic variables, including marital status and educational attainment, differed between groups and could not be fully adjusted for in the regression models because of sample size constraints. This factor raises the possibility of residual confounding, and the observed associations should therefore be interpreted with caution. The study was conducted at a single tertiary centre, which may limit the generalisability of the findings. Future studies with larger, more balanced samples, longer follow-up periods, objective sleep measurements, and data suitable for robust parametric modelling are required to validate and extend the present findings.

## 6. Conclusions

Sleep disturbance is a prevalent but phenotypically heterogeneous contributor to disability in primary headache disorders. The present findings indicate that the sleep domain most strongly associated with headache-related functional burden differs between EM and ECH: daytime sleepiness in EM and global sleep quality combined with sleep hygiene behaviour in ECH. These results support a precision medicine approach to sleep assessment in headache management, whereby the specific sleep domain targeted should reflect the neurobiological characteristics of the individual headache phenotype. Replication in larger, multicentre cohorts is required to validate and extend these observations. Future research should also investigate whether the phenotype-specific sleep–disability relationships identified herein extend to the chronic forms of these disorders—namely, chronic migraine and chronic cluster headache—which carry an even greater burden of sleep disturbance and disability and remain comparatively understudied in this context.

## Figures and Tables

**Table 1:** Sociodemographic characteristics of the study population.

Variable	EM (n = 20)	ECH (n = 21)	HC (n = 18)	*p*-Value
Age (years)	33.0 ± 6.8	32.3 ± 6.8	36.7 ± 7.7	0.164
Female sex, n (%)	14 (70.0)	9 (42.9)	10 (55.6)	0.216
BMI (kg/m^2^)	25.8 ± 5.0	23.8 ± 3.9	24.9 ± 5.1	0.413
Smoking, n (%)	11 (55.0)	11 (52.4)	5 (27.8)	0.182
Alcohol use, n (%)	2 (10.0)	8 (38.1)	5 (27.8)	0.114
Tea/coffee > 500 mL/day, n (%)	11 (55.0)	12 (57.1)	3 (16.7)	0.019 *
Single marital status, n (%)	3 (15.0)	14 (66.7)	10 (55.6)	0.006 *
Currently employed, n (%)	9 (45.0)	17 (81.0)	11 (61.1)	0.050
University education, n (%)	8 (40.0)	9 (42.9)	7 (38.9)	0.003 *

Values are mean ± SD or n (%). Kruskal–Wallis test for continuous variables; chi-squared or Fisher’s exact test for categorical variables. * *p* < 0.05. BMI, body mass index; EM, episodic migraine; ECH, episodic cluster headache; HC, healthy controls.

**Table 2:** Headache characteristics of episodic migraine and episodic cluster headache patients.

Variable	EM (n = 20)	ECH (n = 21)	*p*-Value
Headache days/month	9.3 ± 3.2	6.6 ± 4.3	0.044 *
Disease duration (months)	56.1 ± 89.5	27.4 ± 30.4	0.187
Attack duration (hours)	27.5 ± 31.4	1.8 ± 0.9	<0.001 *
Unilateral pain, n (%)	15 (75.0)	7 (33.3)	0.018 *
Pulsating pain quality, n (%)	14 (70.0)	8 (38.1)	0.083
Stabbing/boring pain quality, n (%)	3 (15.0)	14 (66.7)	0.001 *
Photophobia, n (%)	15 (75.0)	2 (9.5)	<0.001 *
Phonophobia, n (%)	16 (80.0)	2 (9.5)	<0.001 *
Nausea/vomiting, n (%)	16 (80.0)	1 (4.8)	<0.001 *
Cranial autonomic symptoms, n (%)	3 (15.0)	20 (95.2)	<0.001 *
Agitation/restlessness, n (%)	1 (5.0)	12 (57.1)	<0.001 *

Values are mean ± SD or n (%). Mann–Whitney U test for continuous variables; Fisher’s exact test for categorical variables. * *p* < 0.05. EM, episodic migraine; ECH, episodic cluster headache.

**Table 3:** Comparison of sleep, psychological, and disability parameters across groups.

Parameter	EM (n = 20)	ECH (n = 21)	HC (n = 18)	*p*-Value
HIT-6	50.1 ± 6.6	49.7 ± 3.5	—	0.770 ^†^
HADS-Anxiety	13.8 ± 3.9	17.0 ± 2.2	9.2 ± 1.7	<0.001 ^a^
HADS-Depression	15.3 ± 4.3	14.9 ± 3.3	8.0 ± 1.6	<0.001 ^b^
ISI	19.4 ± 3.4	24.7 ± 1.8	6.2 ± 3.0	<0.001 ^a^
ESS	11.1 ± 2.1	16.9 ± 2.6	7.6 ± 3.0	<0.001 ^a^
SHI	21.7 ± 3.8	25.7 ± 3.1	14.3 ± 2.7	<0.001 ^a^
PSQI total	9.2 ± 4.4	9.9 ± 3.0	5.3 ± 2.1	<0.001 ^b^
Component 1 (subjective quality)	1.3 ± 1.0	1.5 ± 1.2	0.2 ± 0.4	<0.001 ^b^
Component 2 (sleep latency)	1.0 ± 1.0	1.6 ± 0.9	0.7 ± 0.5	0.007 ^c^
Component 3 (sleep duration)	1.2 ± 1.1	1.2 ± 0.8	1.0 ± 1.0	0.752
Component 4 (sleep efficiency)	1.2 ± 1.2	0.1 ± 0.5	0.8 ± 1.0	0.002 ^d^
Component 5 (sleep disturbances)	1.7 ± 1.0	1.9 ± 0.7	1.0 ± 0.0	<0.001 ^b^
Component 6 (sleep medication use)	1.4 ± 0.9	1.7 ± 0.8	0.9 ± 0.4	0.007 ^c^
Component 7 (daytime dysfunction)	1.5 ± 1.2	1.8 ± 0.9	0.6 ± 0.5	<0.001 ^b^

Values are mean ± SD. Three-group comparisons: Kruskal–Wallis test with Bonferroni-corrected post hoc pairwise Mann–Whitney U tests. ^†^ HIT-6: EM vs. ECH comparison only (Mann–Whitney U with Bonferroni correction; *p* = 0.770). ^a^ Significant for all three pairwise comparisons (EM–HC, ECH–HC, and EM–ECH). ^b^ Significant for EM–HC and ECH–HC only. ^c^ Significant for ECH–HC only. ^d^ Significant for EM–ECH only. ESS, Epworth Sleepiness Scale; HADS, Hospital Anxiety and Depression Scale; HIT-6, Headache Impact Test-6; ISI, Insomnia Severity Index; PSQI, Pittsburgh Sleep Quality Index; SHI, Sleep Hygiene Index.

**Table 4:** Multivariable linear regression model predicting HIT-6 in episodic migraine (n = 20) and Episodic Cluster Headache (n = 21).

A. Multivariable Linear Regression Model Predicting HIT-6 in Episodic Migraine (n = 20)					
Predictor	B	SE	Β	*p*-Value	95% CI
PSQI	0.005	0.360	0.003	0.990	−0.767, 0.776
ESS	1.633	0.691	0.522	0.033 *	0.150, 3.116
SHI	0.098	0.468	0.057	0.837	−0.905, 1.101
ISI	0.019	0.572	0.010	0.974	−1.207, 1.245
HADS-A	0.660	0.463	0.389	0.177	−0.334, 1.653
**B. Multivariable Linear Regression Model Predicting HIT-6 in Episodic Cluster Headache (n = 21)**					
**Predictor**	**B**	**SE**	**Β**	***p*** **-Value**	**95% CI**
PSQI	0.701	0.207	0.604	0.004 *	0.259, 1.143
ESS	−0.418	0.310	−0.303	0.198	−1.080, 0.243
SHI	0.557	0.238	0.485	0.033 *	0.050, 1.064
ISI	0.583	0.464	0.302	0.228	−0.406, 1.571
HADS-A	−0.277	0.363	−0.170	0.456	−1.051, 0.496

A: Dependent variable: HIT-6 (continuous). Model fit: R^2^ = 0.571; F(5,14) = 3.732; *p* = 0.023. All VIF values < 2.5. * *p* << 0.05. B, unstandardized coefficient; β, standardized coefficient; CI, confidence interval; ESS, Epworth Sleepiness Scale; HADS-A, Hospital Anxiety and Depression Scale—Anxiety subscale; ISI, Insomnia Severity Index; PSQI, Pittsburgh Sleep Quality Index; SE, standard error; SHI, Sleep Hygiene Index. B: Dependent variable: HIT-6 (continuous). Model fit: R^2^ = 0.562; F(5,15) = 3.855; *p* = 0.019. All VIF values < 2.5.

## Data Availability

The data presented in this study are available upon reasonable request from the corresponding author.

## Abbreviations

The following abbreviations are used in this manuscript:

BMI	Body mass index
CI	Confidence interval
EM	Episodic Migraine
ECH	Episodic Cluster Headache
EDS	Excessive Daytime Sleepiness
ESS	Epworth Sleepiness Scale
HADS	Hospital Anxiety and Depression Scale
HC	Healthy Controls
HIT-6	Headache Impact Test-6
ICHD-3	*International Classification of Headache Disorders, Third Edition*
ISI	Insomnia Severity Index
PSQI	Pittsburgh Sleep Quality Index
SHI	Sleep Hygiene Index
STROBE	Strengthening the Reporting of Observational Studies in Epidemiology

## References

[1] Headache Classification Committee of the International Headache Society (IHS) (2018). The International Classification of Headache Disorders, 3rd edition. Cephalalgia.

[2] Woldeamanuel Y.W., Cowan R.P. (2017). Migraine affects 1 in 10 people worldwide featuring recent rise: A systematic review and meta-analysis of community-based studies involving 6 million participants. J. Neurol. Sci..

[3] Ertas M., Baykan B., Orhan E.K., Zarifoglu M., Karli N., Saip S., Onal A.E., Siva A. (2012). One-year prevalence and the impact of migraine and tension-type headache in Turkey: A nationwide home-based study in adults. J. Headache Pain..

[4] Suri H., Ailani J. (2021). Cluster Headache: A Review and Update in Treatment. Curr. Neurol. Neurosci. Rep..

[5] Fischera M., Marziniak M., Gralow I., Evers S. (2008). The incidence and prevalence of cluster headache: A meta-analysis of population-based studies. Cephalalgia.

[6] Lupi C., Evangelista L., Favoni V., Granato A., Negro A., Pellesi L., Ornello R., Russo A., Cevoli S., Guerzoni S. (2018). Rare primary headaches in Italian tertiary Headache Centres: Three year nationwide retrospective data from the RegistRare Network. Cephalalgia.

[7] Husøy A.K., Xu Y.Y., Steinmetz J.D., Aalipour M.A., Aalruz H., Abdulah D.M., Aboagye R.G., Abtahi D., Abu Rumeileh S., GBD 2023 Headache Collaborators (2025). Headache Collaborators. Global, regional, and national burden of headache disorders, 1990–2023: A systematic analysis for the Global Burden of Disease Study 2023. Lancet Neurol..

[8] Steiner T.J., Stovner L.J., Jensen R., Uluduz D., Katsarava Z. (2020). Migraine remains second among the world’s causes of disability, and first among young women: Findings from GBD2019. J. Headache Pain..

[9] Fernández-Ferro J., Ordás-Bandera C., Rejas-Gutiérrez J., Ferro-Rey B., Gómez-Lus S., Láinez Andrés J.M. (2025). The economic burden of migraine: A nationwide cost-of-illness approach from the year 2020 European Health Survey in Spain. Neurologia.

[10] Seng E.K., Martin P.R., Houle T.T. (2022). Lifestyle factors and migraine. Lancet Neurol..

[11] Martin P.R., Reece J., MacKenzie S. (2021). Integrating headache trigger management strategies into cognitive-behavioral therapy: A randomized controlled trial. Health Psychol..

[12] McGeary D.D., McGeary C.A. (2025). Behavioral treatment strategies for headache management. Phys. Med. Rehabil. Clin. N. Am..

[13] Schäfer B. (2024). Non-pharmacological approaches to headache prevention: What is the evidence?. Fortschr. Neurol. Psychiatr..

[14] Domaç F.M., Uludüz D., Özge A., Heinbockel T. (2022). Sleep patterns changes depending on headache subtype and covariates of primary headache disorders. Neurophysiology—Networks, Plasticity, Pathophysiology and Behavior.

[15] Korabelnikova E.A., Danilov A.B., Vorobyeva Y.D., Latysheva N.V., Artemenko A.R. (2020). Sleep disorders and headache: A review of correlation and mutual influence. Pain Ther..

[16] Ferini-Strambi L., Galbiati A., Combi R. (2019). Sleep disorder-related headaches. Neurol. Sci..

[17] Almansour N.A., Alsalamah S.S., Alsubaie R.S., Alshathri N.N., Alhedyan Y.A., Althekair’s F.Y. (2025). Association between migraine severity and sleep quality: A nationwide cross-sectional study. Front. Neurol..

[18] Tiseo C., Vacca A., Felbush A., Filimonova T., Gai A., Glazyrina T., Hubalek I.A., Marchenko Y., Overeem L.H., Piroso S. (2020). Migraine and sleep disorders: A systematic review. J. Headache Pain..

[19] Barloese M.C.J. (2015). Neurobiology and sleep disorders in cluster headache. J. Headache Pain..

[20] Burish M., Kim S.A., Ran C., Yoo S.-H., Lee M.J., Belin A.C. (2025). Reviewing the complex relationship between circadian rhythms and cluster headache. Cephalalgia.

[21] Pilati L., Torrente A., Alonge P., Vassallo L., Maccora S., Gagliardo A., Pignolo A., Iacono S., Ferlisi S., Di Stefano V. (2023). Sleep and chronobiology as a key to understand cluster headache. Neurol. Int..

[22] Barloese M.C.J. (2021). Current understanding of the chronobiology of cluster headache and the role of sleep in its management. Nat. Sci. Sleep.

[23] Benkli B., Kim S.Y., Koike N., Han C., Tran C.K., Silva E., Yan Y., Yagita K., Chen Z., Yoo S.-H. (2023). Circadian features of cluster headache and migraine: A systematic review, meta-analysis, and genetic analysis. Neurology.

[24] Daou M., Vgontzas A. (2024). Sleep symptoms in migraine. Curr. Neurol. Neurosci. Rep..

[25] Gorgoni M., Giuliani G., Fratino M., Di Piero V. (2024). The objective assessment of sleep in cluster headache: State of the art and future directions. J. Sleep Res..

[26] Duan S., Ren Z., Xia H., Wang Z., Zheng T., Liu Z. (2022). Association between sleep quality, migraine and migraine burden. Front. Neurol..

[27] Donnet A., Lanteri-Minet M., Guegan-Massardier E., Mick G., Fabre N., Géraud G., Lucas C., Navez M., Valade D. (2007). Chronic cluster headache: A French clinical descriptive study. J. Neurol. Neurosurg. Psychiatry.

[28] Ray J.C., Stark R.J., Hutton E.J. (2022). Cluster headache in adults. Aust. Prescr..

[29] Sun Y., Laksono I., Selvanathan J., Saripella A., Nagappa M., Pham C., Englesakis M., Peng P., Morin C.M., Chung F. (2021). Prevalence of sleep disturbances in patients with chronic non-cancer pain: A systematic review and meta-analysis. Sleep Med. Rev..

[30] Debta D.K., Padarabinda Tripathy K., Jain T., Behera P.K., Chodey S., Sai M., Agarwal P., Karnati B.V., Padhan P. (2025). Prevalence of Sleep Disturbances in Rheumatoid Arthritis and Its Association With Disease Severity: A Hospital-Based Cross-Sectional Observation. Cureus.

[31] Dikmen P.Y., Bozdağ M., Güneş M., Koşak S., Taşdelen B., Uluduz D., Ozge A. (2020). Reliability and validity of Turkish version of Headache Impact Test (HIT-6) in patients with migraine. Noro Psikiyatr. Ars..

[32] Aargün M.Y., Kara H., Anlar Ö. (1996). Pittsburgh Uyku Kalitesi İndeksinin geçerliği ve güvenirliği. Türk. Psikiyatr. Dergisi..

[33] Boysan M., Güleç M., Beşiroğlu L., Kalafat T. (2010). Uykusuzluk Şiddetİndeksi’nin Türk örneklemindeki psikometrik özellikleri. Anadolu Psikiyatr. Dergisi..

[34] Izci B., Ardic S., Firat H., Sahin A., Altinors M., Karacan I. (2008). Reliability and validity studies of the Turkish version of the Epworth Sleepiness Scale. Sleep Breath.

[35] Ozdemir P.G., Boysan M., Selvi Y., Yildirim A., Yilmaz E. (2015). Psychometric properties of the Turkish version of the Sleep Hygiene Index in clinical and non-clinical samples. Compr. Psychiatry.

[36] Aydemir O., Guvenir T., Kuey L., Kultur S. (1997). Validity and reliability of Turkish version of Hospital Anxiety and Depression Scale. Turk. Psikiyatr. Derg..

[37] Faul F., Erdfelder E., Lang A.G., Buchner A. (2007). G*Power 3: A flexible statistical power analysis program for the social, behavioral, and biomedical sciences. Behav. Res. Methods.

[38] Benbir G., Karadeniz D., Göksan B. (2012). The characteristics and subtypes of headache in relation to age and gender in a rural community in Eastern Turkey. Agri.

[39] Kurt S., Kaplan Y. (2008). Epidemiological and clinical characteristics of headache in university students. Clin. Neurol. Neurosurg..

[40] Tuncer Issı Z., Akbulut N., Öztürk V. (2022). Cluster headache: A single tertiary center study. Neurol. Res..

[41] Rozen T.D. (2015). Emergency Department and Inpatient Management of Status Migrainosus and Intractable Headache. Continuum.

[42] Coppola G., Arruda M.A., Ashina M., Barloese M., Belin A.C., Bottiroli S., Chowdhury D., De Icco R., Di Lorenzo C., Di Stefano G. (2025). Hallmarks of primary headache: Part 3—cluster headache. J. Headache Pain..

[43] Goadsby P.J., Holland P.R., Martins-Oliveira M., Hoffmann J., Schankin C., Akerman S. (2017). Pathophysiology of migraine: A disorder of sensory processing. Physiol. Rev..

[44] Wei D.Y., Yuan Ong J.J., Goadsby P.J. (2018). Cluster headache: Epidemiology, pathophysiology, clinical features, and diagnosis. Ann. Indian Acad. Neurol..

[45] Ikram W. (2025). The correlation between migraine frequency and sleep disturbances in adults: A cross-sectional study. Cureus.

[46] Jennysdotter Olofsgård F., Ran C., Spulber S., Steinberg A., Sjöstrand C., Waldenlind E., Lantz M., Sundholm A., Söderström M., Dahlgren A. (2025). Characterization of insomnia and sleep quality in a cohort with cluster headache. Headache.

[47] Saçmacı H., Tanik N., İnan L.E. (2022). Current perspectives on the impact of chronic migraine on sleep quality: A literature review. Nat. Sci. Sleep.

[48] Cho S., Lee M.J., Park H.R., Kim S., Joo E.Y., Chung C.S. (2020). Effect of sleep quality on headache-related impact in primary headache disorders. J. Clin. Neurol..

[49] de Coo I.F., van Oosterhout W.P.J., Wilbrink L.A., van Zwet E.W., Ferrari M.D., Fronczek R. (2019). Chronobiology and sleep in cluster headache. Headache J. Head Face Pain.

